# A Framework-Based Approach to Assessing Mental Health Impacts of the COVID-19 Pandemic on Children and Adolescents

**DOI:** 10.3389/fpsyt.2021.655481

**Published:** 2021-05-13

**Authors:** Ping-I Lin, Gautam Srivastava, Linda Beckman, Yunhwan Kim, Maria Hallerbäck, Drew Barzman, Michael Sorter, Valsamma Eapen

**Affiliations:** ^1^School of Psychiatry, University of New South Wales, Sydney, NSW, Australia; ^2^South Western Sydney Local Health District, Liverpool, NSW, Australia; ^3^Department of Medical Biochemistry and Microbiology, Uppsala University, Uppsala, Sweden; ^4^Department of Health Sciences, Karlstad University, Karlstad, Sweden; ^5^Centre for Child and Adolescent Mental Health, Karlstad University, Karlstad, Sweden; ^6^Region Värmland, Karlstad, Sweden; ^7^Division of Child and Adolescent Psychiatry, Cincinnati Children's Hospital Medical Center, Cincinnati, OH, United States

**Keywords:** COVID-19, mental health, children, adolescents, anti-contagion policy

## Abstract

The COVID-19 pandemic has yielded extensive impacts globally in the year of 2020. Although the mental health of children and adolescents may be particularly susceptible to stressors stemming from the pandemic and anti-contagion policies, most ongoing efforts are geared toward curbing the viral spread. In the current perspective, we have identified four domains of factors corresponding to an ecological framework that may directly or indirectly influence the mental health of children and adolescents during the pandemic. The evidence suggests that anti-contagion policies might trigger cascades that impact the mental health of children and their families through multiple different sectors that used to form a safety net for youths. Additionally, children with neuropsychiatric disorders could experience exacerbated symptoms during the pandemic. Furthermore, the risk of domestic violence has surged during the pandemic, which further compounds the imminent mental health crisis. A mental health pandemic could be inevitable if no proactive prevention strategies were in place. Therefore, we recommend understanding each individual mental health risk pathway via the ecological framework in order to develop integrative prevention and intervention strategies.

## Introduction

Concerns about the impact of coronavirus disease 2019 (COVID-19) on a vulnerable population, such as children and adolescents, have been increasingly noted around the world. Anti-contagion measures to curb the spread of SARS-CoV-2 virus, such as school closures, lockdowns, and travel restrictions, have led to changes that disrupt daily routines and resources, which in turns could impact mental health of children and adolescents. From the biological perspective, the SARS-CoV-2 virus can severely disrupt brain function via immune responses leading to a cytokine storm (1). Recent evidence also demonstrates that some genetic networks may play a role in both COVID-19 and psychiatric disorders such as post-trauma stress disorder, bipolar disorder, and schizophrenia (2). From the psychological perspective, the association between emotional reaction such as loneliness and depression in children and adolescents, has been reported (3). Little is known about how the COVID-19 pandemic plays a role in the mental health of children and adolescents.

**Figure 1:**
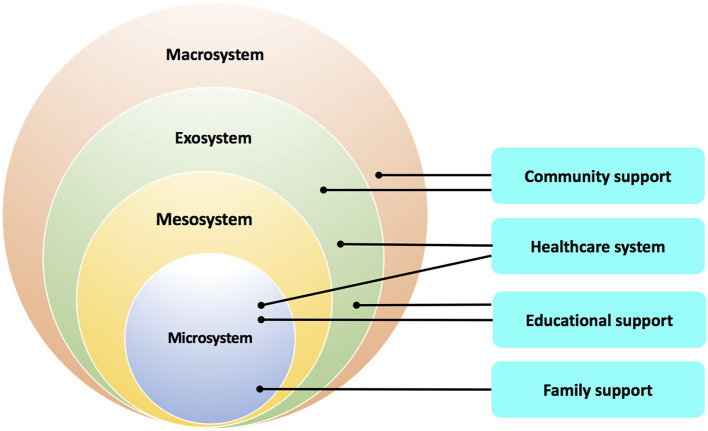
The domains of factors impacted by the COVID-19 pandemic corresponding to the four levels of the ecological framework are shown. Each domain could be involved in more than one level of the ecological framework.

In the current review, we discuss four domains of factors relevant to the mental health of children and adolescents: (1) educational support, (2) health care system, (3) community support, and (4) family support. The elements in these four domains can be classified into higher-demand (e.g., the need for more technological support for online learning and telemedicine) vs. lower-resource (e.g., less financial support due to parental unemployment) components affected by the pandemic (Table 1). How these four domains interact with each other to influence mental health of children and adolescents during and after the COVID-19 pandemic can be examined using the Bronfenbrenner's ecological framework (4). This ecological system includes: (1) the *microsystem*, in which the individual can directly interact with its components, such as family members and peers, (2) the *mesosystem*, which involves the interaction of two, or more components in the microsystem (e.g., interplay between school, family, and healthcare providers), (3) the *exosystem*, which exerts more distal or indirect effects on the individual (e.g., parents' experience, etc.), and (4) the *macrosystem* consisting of those components that determine how public affairs are operated (e.g., economic condition of the society)—which can impact the individual well-being via collective environmental effects. How the four domains correspond to the different levels of the ecological framework is illustrated in Figure 1. This ecological framework can provide a theoretical ground for the relationship between these four domains and why they should be integrated into the modified anti-contagion measures should the similar event occur in the future. Furthermore, an understanding of the interplay between these components in the four domains can inform researchers and clinicians of how to evaluate mental health issues among children and adolescents to formulate prevention/intervention strategies accordingly.

**Table 1:** Factors in the four domains classified into “higher demands” vs. “lower resources” categories are shown.

**Domain**	**Higher demands**	**Lower resources**
School	Technological support for online learning	Face-to-face interactive learning
		Peer support
		Special educational support
		School mental health counseling
		Sports activities at school
Family	Mental health support for parents	Household financial stability
	Technological support for online learning	Outdoor family activities
	Interventions implemented by parents	
Healthcare system	Telemedicine	Face-to-face healthcare service
Community	Financial support	Interactive social events
		Freedom to travel
		Community-based support

## Educational Support: Impact of School Closures and Online Learning on Mental Health

One of the non-pharmacological interventions (NPIs) that aim to slow down the spread of infections, such as influenza, is school closures (5–8). Approximately 80–90% of children experienced school closures during the COVID-19 pandemic around the world in 2020 (9, 10). However, the effect of school closures on the spread of COVID-19 remains debatable since children exposed to the SARS-CoV-2 virus may be asymptomatic or show minimal symptoms, and given the fact that basic reproduction number (R_0_) for the spread of COVID-19 among school-aged children in a classroom has been estimated to be <1 (11). However, school closures may adversely impact the well-being of children (12), and may reduce the availability of health-care workforce, which in turn can negatively impact health outcomes (13). A recent modeling study suggests that school closures can only reduce mortality rates by 2–4% (14). However, this reduction in mortality rates was estimated based on the assumption that school closures were the only intervention implemented in a society, which is not the case in the vast majority of countries (15).

Emerging literature has addressed the concern about the detrimental impact of school closures on mental health among children and adolescents. These concerns may fall into two categories: changes in lifestyle and exacerbation of existing vulnerability. School closures impose restrictions on two types of activities that are critical for favorable mental health among children and adolescents: social and physical activities. Building, maintaining, and enjoying social connections with peers form a critical developmental task during childhood and adolescence (16), which exert a significant impact on their mental health (17). Schools can provide ample opportunities for social connections, which are severely, if not entirely, jeopardized by school closures due to the COVID-19 pandemic (18). The second type of activities relates to physical activities. A review study presents a consistent relationship between physical activity and mental health among children and adolescents (19). For example, school closures during the COVID-19 pandemic not only decrease the time for established physical activities, but also increase time and opportunities for sedentary behaviors (20). Indeed, one empirical study on Italian children and adolescents with obesity reports that, 3 weeks after COVID-19 lockdown, the participants spent significantly less time on sports (i.e., 2.30 h less per week) while their screen time increased significantly (i.e., 4.85 h more per week) (21). Another empirical study on children and adolescents in Italy and Spain reported on the parental perception that, as a result of the COVID-19 quarantine, their children experienced a range of emotional difficulties and more than half of their children spent <30 min per day on physical activities (22). Schools can provide augmenting support for students' difficulties in various contexts (9, 10). Multiple layers of socializing agents influence a child's development, and schools and teachers are one of the most influential socializing agents for children and adolescents (23). When children and adolescents do not receive proper care from their guardians, and/or when they are under certain risks at home, schools, and teachers can serve as a safeguard and buffer against potential negative consequences (24). For example, schools can be a haven for the students who suffer from abuse or neglect (25). Schools provide support for leisure activities for the students who cannot afford them due to financial hardships (26). In addition, schools can provide mental health counseling for children and adolescents in need (27). School closures during the COVID-19 pandemic have caused a hiatus in resources that youths would otherwise benefit from Lee (9), Van Lancker et al. (10), and Golberstein et al. (28).

The mental health of children with special educational needs (e.g., individuals with neurodevelopmental disorders) as well as their caretakers is also impacted by the pandemic. In a study by Asbury et al., parents of children (5–18 years old) with Special Educational Needs and Disabilities in the UK were asked to describe the impact of COVID-19 on their mental health and that of their child (29). Both parents and children were found to be experiencing loss, worry and changes in mood, and behavior as a result of the rapid social changes that have occurred. Some parents reported feeling overwhelmed and experienced stress to enhance a child's understanding and awareness of personal health and safety. Compared to their neurotypical peers, distant learning programs appear to be more challenging for these children.

Although technology with a focus on digital tools has become an essential strategy for learning activities during the pandemic, it also poses challenges for some individuals. For example, the internet speed may not accommodate with online meetings when there are multiple users at the same household. Online learning may also make it more challenging to be actively engaged in either lectures or interactive discussions and affect their learning outcomes. As Drane and colleagues stated, children living in financially disadvantaged communities could be subject to long-term educational disengagement, digital exclusion, poor technology management, and increased psychosocial challenges during the pandemic (30). These challenges, partly due to family's capacity of resources and partly due to individual reasons, could trigger more stress for students as well as parents/carers.

## Health Care System

### Impact of The Overwhelmed Medical Care System on Mental Health Care Quality

The health care systems have been overwhelmed by the near impossibility of responding to the needs of a multitude of seriously infected patients (31). Since the World Health Organization (WHO) declared a public health emergency on January 30, 2020 (WHO, 2020), relentless efforts have been made to contain the pandemic, which has included treating innumerable patients with limited resources and efforts to find effective treatments such as a vaccine or other drugs, all of which have overwhelmed the medical care system unprecedentedly (32). This has caused gross neglect of other health priorities, such as care of chronic diseases, most notably, mental health care. Paradoxically, the importance of the latter has increased several times, further compounded by the negative impact of the containment measures taken to subdue the virus.

For children or adolescents with neuropsychiatric disorders or any mental health issues, changes in modes of or access to mental health care may be more disruptive than their unaffected peers. Closures of schools and outpatient units, restrictions of hospital policies, along with decreased attention from parents amidst the pandemic, might exacerbate existing neuropsychiatric conditions of affected children (28). Visiting physicians regularly, acquiring prescribed medications, and visits at emergency departments, have become much more difficult. Some children or adolescents with psychiatric disorders are contracted with COVID-19 because these patients may present with impulsivity, irritability, or poor judgement due to cognitive impairments, which may adversely impact personal hygiene behaviors to increase the risk of infections (33, 34). Previous evidence indicates that severely mentally ill patients have an increased risk of pneumonia, thus making the present pandemic more severe and fatal for them (35). Additionally, individuals with intellectual disability are found to have higher mortality rates associated with COVID-19 (36), which may be partially attributable to feeding difficulty due to deteriorating muscle tone and behavioral dysregulation (37) and challenges in engaging them in proper health care. These findings have lent support to the hypothesis postulated by Sartorius that mental health comorbidities to other infectious diseases will be the main challenge for medicine in the twenty-first century (38).

One recent study observed exacerbated mood and behavioral symptoms in children with psychiatric diagnoses, such as attention deficit and hyperactivity disorder (ADHD) during the pandemic (39). According to this study based on parental reports, ADHD behaviors were significantly worsened in comparison to their normal state. Also, the children's negative mood state was associated with ADHD symptoms. The authors further found that a parent's mood state also had an impact on children's ADHD symptom. Therefore, the pandemic-associated challenges might also increase stress levels of parents, which in turn affected how parents handled ADHD symptoms of children.

Choi et al. observed that the unprecedented global health scenario created by the COVID-19 pandemic has ushered in a “Second Pandemic” of the mental health crisis in the global health system and community (40). The latter will substantially affect all children, adolescents, adults, old people, and thus has to be addressed simultaneously, despite a highly overwhelmed medical care system. Dalton et al. found that healthcare workers are experiencing an unprecedented demand for caring for a predominantly adult patient population, thus magnifying the invisibility of children's urgent psychological needs who account for over 42% of the world's population (41).

### Impact of Access to Mental Health Services

School closures are likely to put some of the school-based mental health programs on hold. Children needing treatments may have to seek support from Emergency Departments or clinicians at private clinical settings—who may have no prior knowledge or access to the relevant information including reports from schools, due to privacy regulations, and thus may not be able to provide optimal support for the school-aged children (28). For children and adolescents with ADHD, the changes in their daily life due to restrictions may be challenging. The stress may lead to more behavioral problems. Further, since schools are closed, students may need to organize their studies and attend online classes which they may find difficult to manage due to poor organizational ability, distractibility, and poor focus. Hence professional bodies such as the European ADHD Guidelines Group (EAGG) has published guidance on the assessment and management of ADHD during the COVID-19 virus pandemic (42). The EAGG are recommending that pharmacological treatment of ADHD should continue as usual. Further, individuals should, if indicated, be offered the opportunity to start medication. Telephone and appropriate online video technology should be used and routine cardiovascular clinical examination and face-to-face visits can be postponed. To reduce oppositional defiant and disruptive behaviors, families are recommended to use behavioral parenting strategies.

Another group of children that may also be substantially impacted by the pandemic are those diagnosed with autism spectrum disorder (ASD). The impact of the pandemic is attributable not only to disruptions of in-person health service but also the core features of ASD: an obsession with routine (43). A recent parent survey shows that more than three-fourths of parents felt that managing ASD children's behavioral disturbances became more difficult amidst the pandemic than the pre-outbreak period. Additionally, the survey reveals that at least one in every three children has presented with more frequent or more intense behavioral problems (44).

## Community Support: Socioeconomic Challenges Imposed By The Pandemic

Stressors such as inadequate information, lack of in-person contact with classmates, friends, and teachers, lack of personal space at home, economic downturn, and family financial loss, increased alcohol consumption in the household, can all have problematic and enduring effects on children and adolescents than the infection itself (28, 45). The COVID-19 pandemic may hence worsen existing mental health problems and lead to more cases among children and adolescents because of the combination of different stressors. For example, learning from previous pandemics, Sprang and Silman found that the mean post-traumatic stress scores were four times higher in children who had been quarantined than in those who were not quarantined (46). Furthermore, the interaction between lifestyle changes and psychosocial stress caused by home confinement could further aggravate the detrimental effects on child physical and mental health, which could cause a vicious circle (47).

In the event of home confinement, parents are often the closest and best resource for children. Close and open communication with children is the key to identifying any physical and psychological issues and comforting children in prolonged isolation. Children are constantly exposed to epidemic-related news, so having direct conversations with children about these issues could alleviate their anxiety (48). However, parents of children who are hospitalized for COVID-19 experience a great deal of stress, depression, and anxiety (48). These phenomena reflect a dearth of community-based resources to ameliorate mental health impacts due to extensive anti-contagion policies. Establishing community-based resources amidst the pandemic requires timely efforts of linking different sectors, such as school-based and health care services as well as governmental agencies to provide educational, health, and financial supports for affected individuals, particularly for those from disadvantaged populations.

## Family Support

### Mental Health of Parents or Caretakers

Family support has been impacted by the pandemic because of the mental health of parents (or caretakers). In a study by Yuan et al. (48), 50 parents of children hospitalized during the COVID-19 epidemic (EH), and 50 parents of children hospitalized during the non-epidemic period (NEH) were included and scores for anxiety, depression, and dream anxiety were compared. The anxiety score of EH parents was significantly higher than that of parents of NEH children. Similarly, the depression scores of parents of EH children were also significantly higher than that of NEH children. Simultaneously, the VDAS scores and SF-36 scores of parents of EH children were significantly higher than that of NEH children. The mental health problems of parents of EH children were more serious, and their anxiety and depression were more severe, than the mental health problems encountered by parents of NEH children.

An alarming trend of the adverse consequence of the pandemic is domestic violence, which is associated with a range of factors including economic stress, disaster-related instability, increased exposure to exploitative relationships, and reduced options for support (49). Recently, anecdotal evidence from several countries has shown increases in intimate partners, women and violence toward children due to isolation and quarantine. Furthermore, already vulnerable children with special needs might experience such impacts much more acutely than the general child population (50). The increasing domestic violence may be attributable to stress associated with financial constraints and health concerns, which can be exacerbated by increasing substance misuse during the COVID-19 pandemic (51, 52).

### Mental Health of Children and Adolescents

Pisano et al. using a questionnaire study with 6,510 children (4–10 years old), reported that one in four children (26.48%), showed regressive symptoms such as the demand for physical proximity to their parents during the night and almost one in five (18.17%) manifested fears that they never had before (53). Half of the children (53.53%) showed increased irritability, intolerance to rules, whims and excessive demands, and one in five presented with mood changes (21.17%) and sleep problems including difficulty falling asleep, agitation, and frequent waking up (19.99%). One in three (34.26%) displayed nervousness about the topic of the pandemic when it was mentioned at home or on TV. Almost one in three (31.38%) seemed calmer and one in two (49.57%) seemed wiser and more thoughtful. Almost all (92.57%) seemed able to adapt to the pandemic restrictions; even though one in two (43.26%) seemed more listless in terms of the activities they used to perform before the pandemic including playing, studying, and gaming.

Orgiles et al. surveyed 1,143 parents of Italian and Spanish children aged 3–18 years, providing information about how the quarantine affects their children and themselves, compared to before the home confinement (22). The results showed that 85.7% of the parents perceived changes in their children's emotional state and behaviors during the quarantine. The most frequent symptoms were difficulty concentrating (76.6%), boredom (52%), irritability (39%), restlessness (38.8%), nervousness (38%), feelings of loneliness (31.3%), uneasiness (30.4%), and worries (30.1%). As expected, children of both countries used monitors more frequently, spent less time doing physical activity, and slept more hours during the quarantine. Furthermore, parents tended to report more emotional problems in their children when they spend less time together compared to other families.

## Discussions

The ecological framework can be useful in expanding the scope of research questions and making cross-disciplinary connections. Consistent with an ecological perspective, our review of research addresses key characteristics of children and adolescents in the COVID-19 pandemic. These four domains, which include family, school, healthcare, and community, contains the most essential components that can affect or reflect the pandemic's impact on mental health issues of children and adolescents. The ecological framework allows us to examine the relationship between these components/domains, which can help clinicians identify predisposing, precipitating, and perpetuating factors, associated with the pandemic and strategize intervention and intervention plans accordingly.

The recommendations based on this framework are composed of two steps of information processing and evaluation. *Step one* is to categorize potential risk factors associated with mental health issues. As mentioned earlier, the components associated with mental health issues in the four domains affected by the pandemic can be classified into “higher-demand” vs. “lower-resource” categories. Higher-demand components may not only require an increased amount of existing resources but also depend on new solutions to fulfill the need. Similarly, lower-resource components may require alternative sources to fill need gaps. Table 1 provides a template for the risk-assessment matrix for clinicians. This approach can allow us to identify and prioritize points of intervention (or even prevention). *Step two* is to understand the “risk pathway” (i.e., how sequential events or responses that lead to mental health consequences) through the conceptual ecological framework consisting of microsystem, mesosystem, exosystem, macrosystem, and, finally, chronosystem.

Children and adolescents, particularly those with neuropsychiatric conditions, may provide an example of how the ecological framework can be used to understand the “mental health risk pathway” in the context of COVID-19 pandemic. Their vulnerability can be attributable to limited access to support from communities due to anti-contagion policies and overwhelmed health care systems. Simultaneously, families are dealing with further challenges due to self-isolation or quarantine such as parents “working from home,” physical space limitations, or having to care for family members who may have acquired the infection or are at high risk for COVID-19 infection etc. Other stressors may include fear of infection, limited access to health care, inadequate food and medical supplies, loss of employment and financial problems, relationship stress, to name a few of them. Furthermore, parental stress can negatively influence parenting ability and may lead to domestic violence or other maladaptive coping which in turn can negatively impact the parent-child relationship and interactions. This scenario exemplifies how microsystem (e.g., parents under stress), mesosystem (e.g., reduced support from collaborations between school and family), and macrosystem (e.g., economic downturn leading to unemployment of parents), could interact with each other form a vicious cycle. Finally, how chronosystem (i.e., the effect of time) may be changed as the pandemic unfolds and universal vaccination is underway should be continuously monitored.

These stressors need to be ameliorated by cross-disciplinary strategies governed by integrative policies. It is hence critical to provide mental health care service to simultaneously address the concerns of both children and their families and implement strategies to proactively engage multiple stakeholders. A better understanding of the relationship between these stressors (e.g., either linear, circular, or reciprocal relationship) can help us identify the point of intervention and generate novel hypotheses to delineate mental health risk pathways. Such strategies may include the collaborations between clinical settings, community-based agencies that provide recourses for individuals and families, and school-based support systems, to form special task forces that can coordinate trans-institutional supports. Specifically, novel telemedicine platforms that can enhance and support patients' adherence to interventions despite the anti-contagion policies and enhance cross-talks between different resources have become increasingly important.

To sum up, the causes of impacts of the COVID-19 pandemic on the mental health of children and adolescents are manifold. Therefore, an integrative approach is needed to tackle these issues with joint efforts to increase the resilience of youths and society as a whole. Recent evidence suggests that the multiple waves of this pandemic might become inevitable until vaccination can effectively keep the large-scale viral spread under control. Therefore, how to integrate these cross-disciplinary strategies into mental health for children and adolescents via the ecological framework shall be established as a long-term implementation plan.

## Data Availability Statement

The original contributions presented in the study are included in the article/supplementary material, further inquiries can be directed to the corresponding authors.

## Author Contributions

P-IL and VE were in charge of the conceptualization process for this manuscript. All authors contributed to the article and approved the submitted version.

## Conflict of Interest

The authors declare that the research was conducted in the absence of any commercial or financial relationships that could be construed as a potential conflict of interest.
